# Supplementary material for: Sex-Specific Effects of Long-Term Antipsychotic Drug Treatment on Adipocyte Tissue and the Crosstalk to Liver and Brain in Rats
Source: Int J Mol Sci. 2024 Feb 11;25(4):2188. doi: 10.3390/ijms25042188 (PMC10889281; doi:10.3390/ijms25042188)

Supplement

Figure S1

a. Chemiluminescence detection of the membranes incubated with the primary antibodies against PerilipinA, HSL, hepcidin, Glut4 ande IRß and the respective secondary antibodies, (HRP-conjugated).

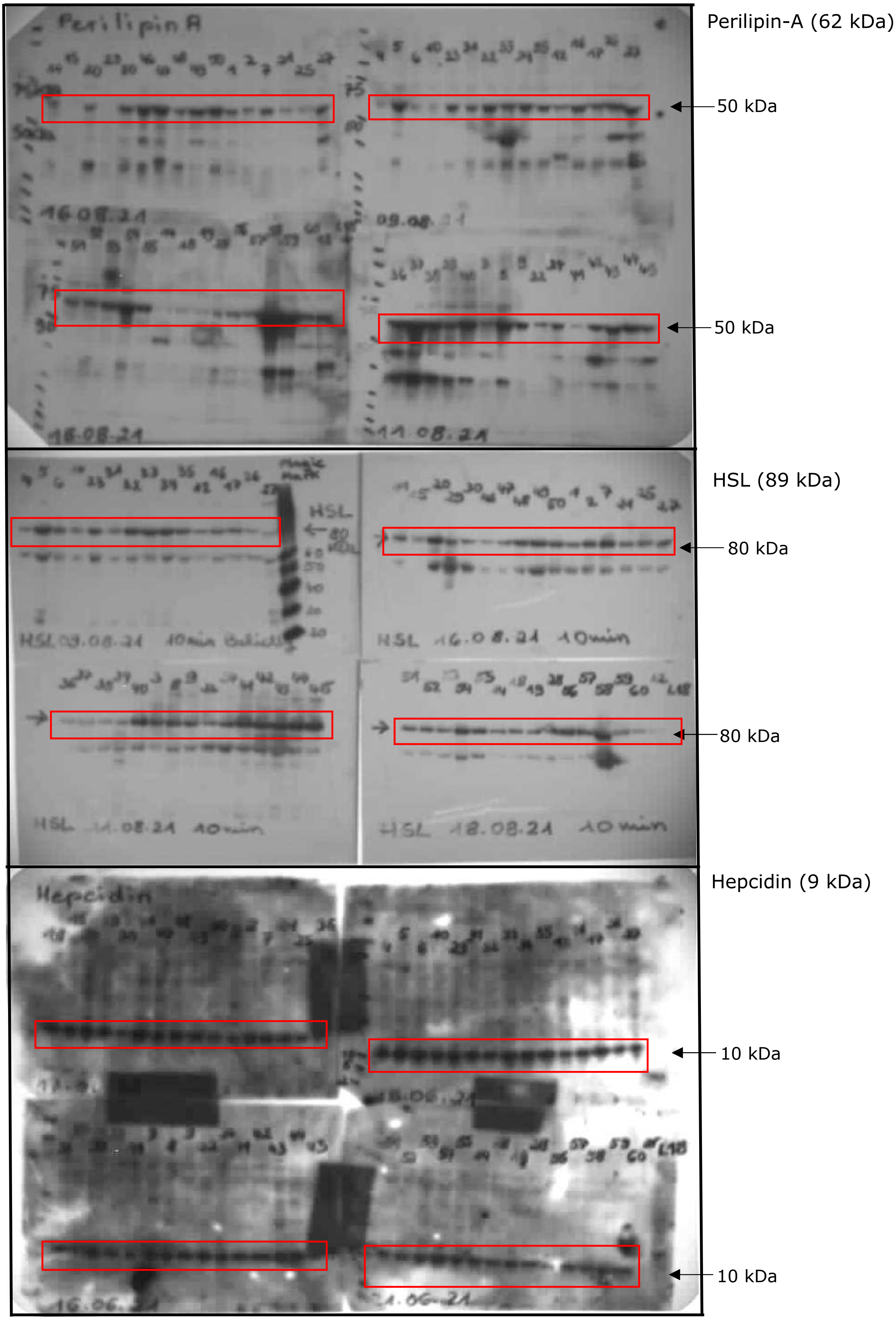

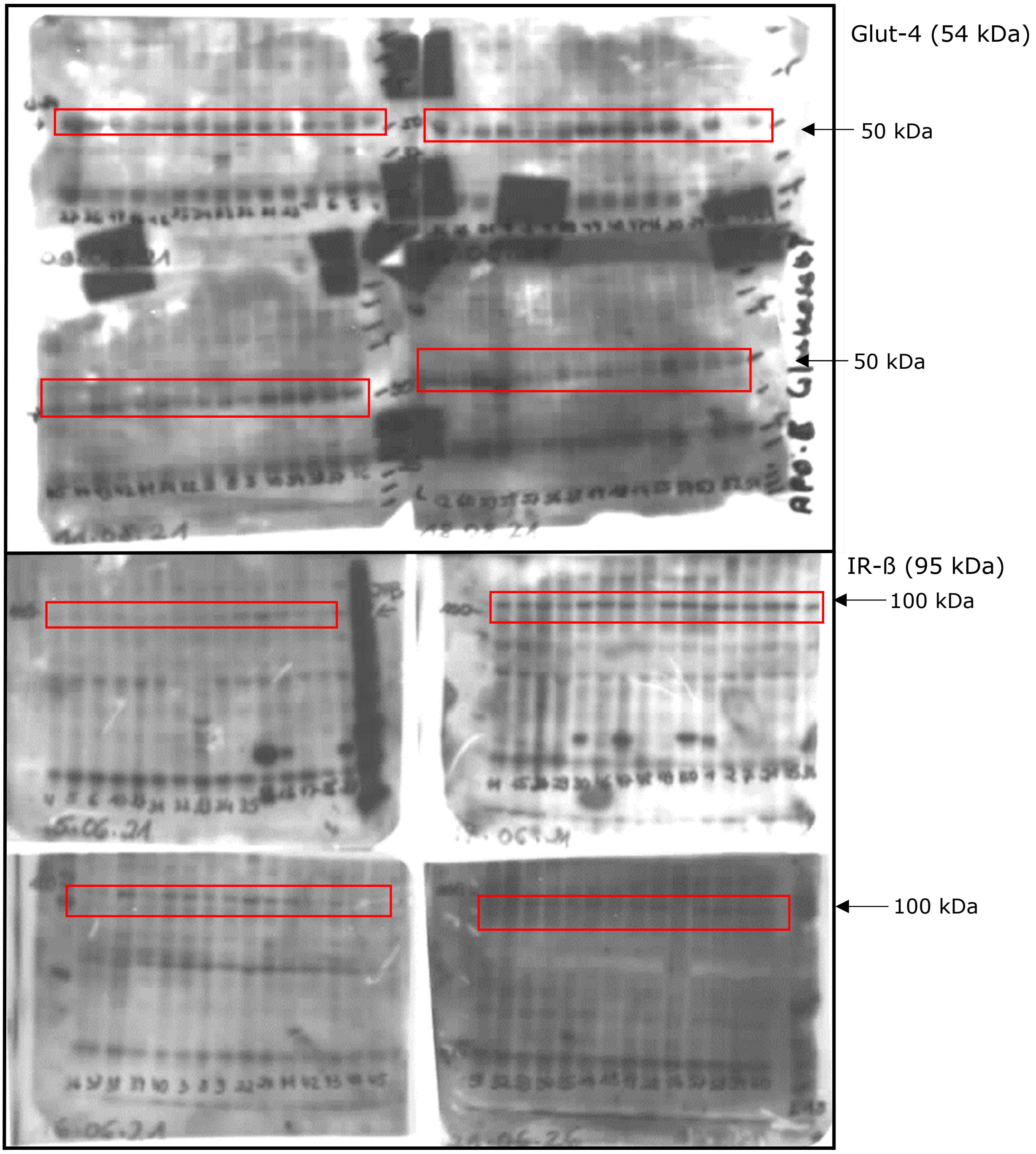

Glut-4 (54 kDa)
50 kDa
50 kDa
IR-ß (95 kDa)
100 kDa
100 kDa

b. Westernblot membranes stained with Ponceau red

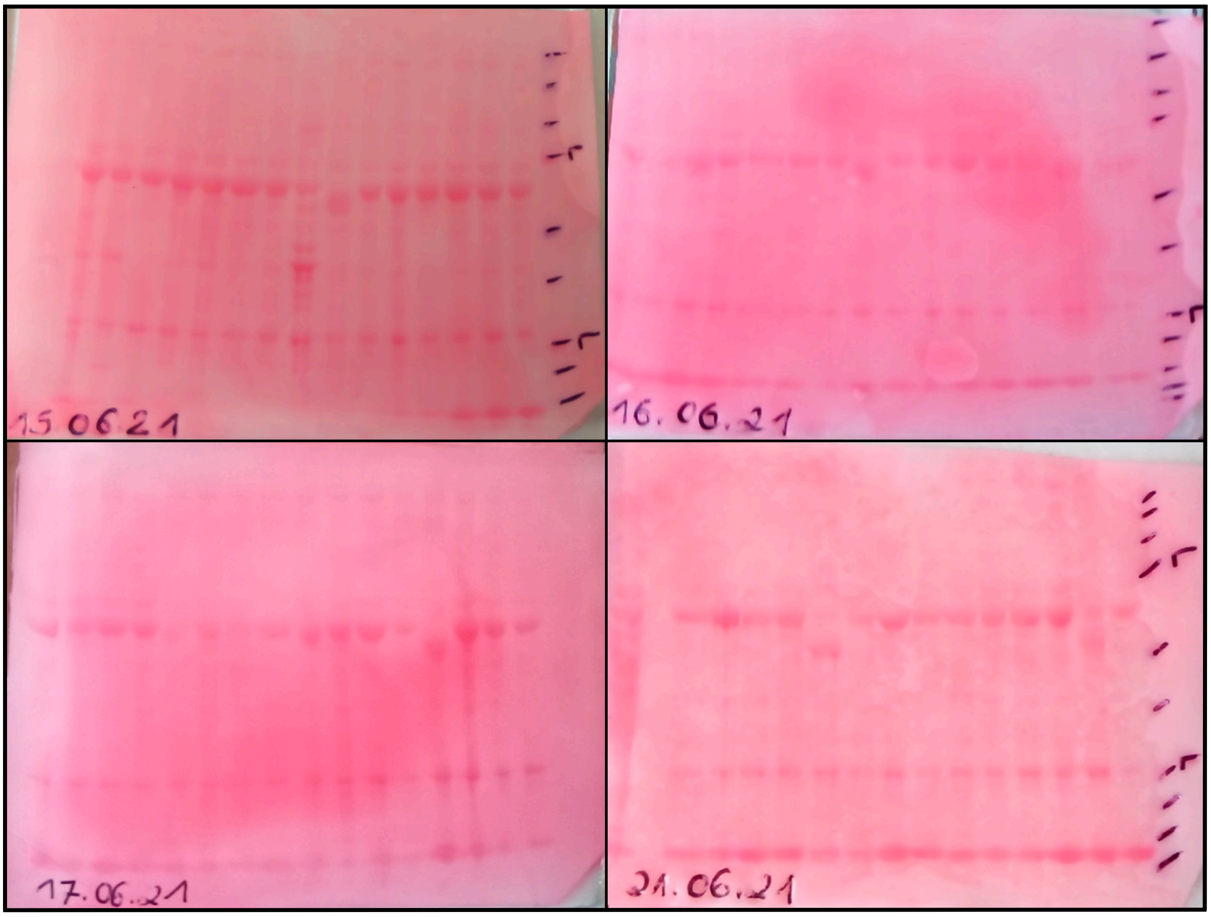

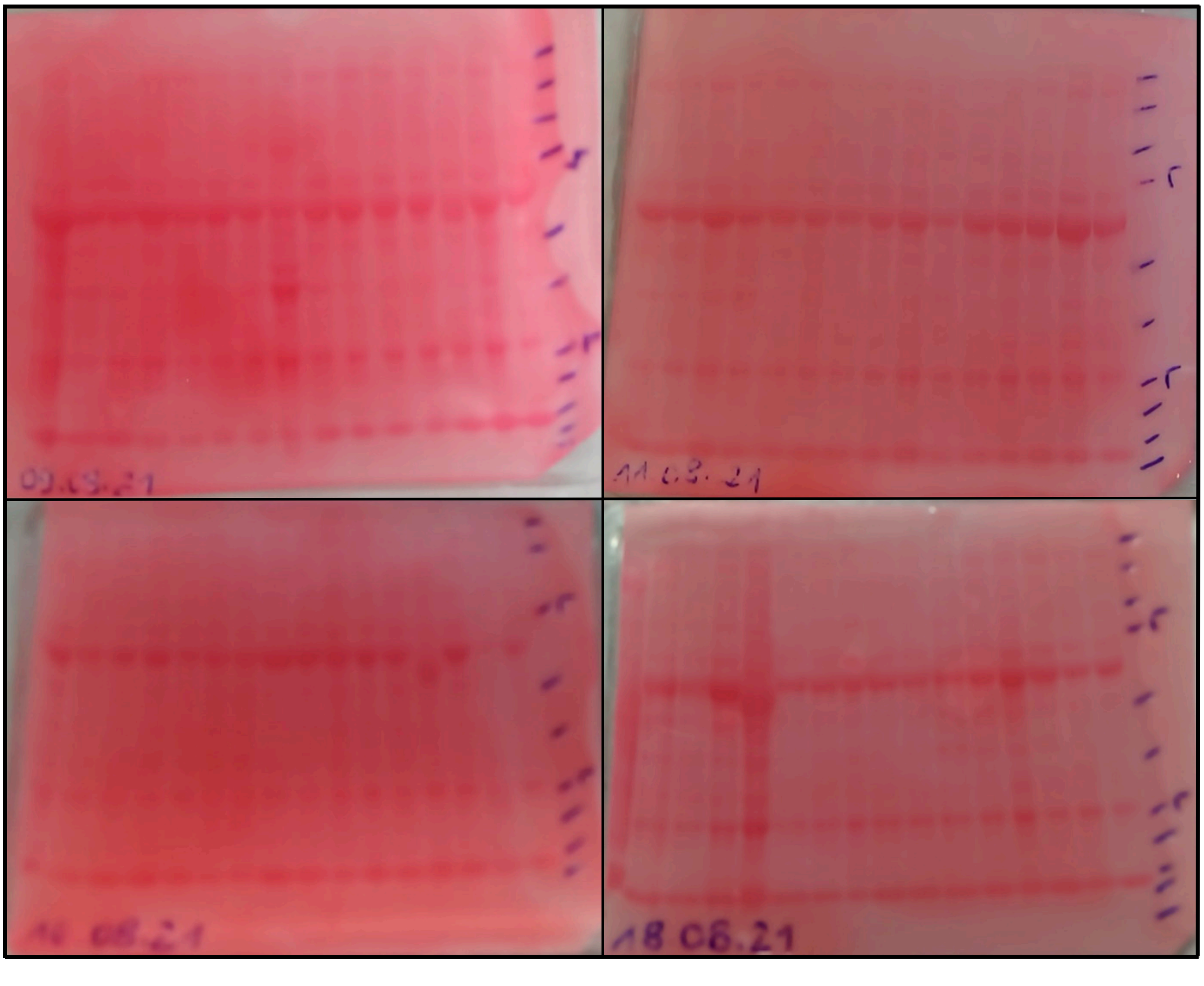

Figure S2

A.-D. Nonparametric correlation of protein level of leptin (abscissa) and percentage adipose tissue mass (ordinate) for male and female controls, and male and female haloperidol medicated SD rats.

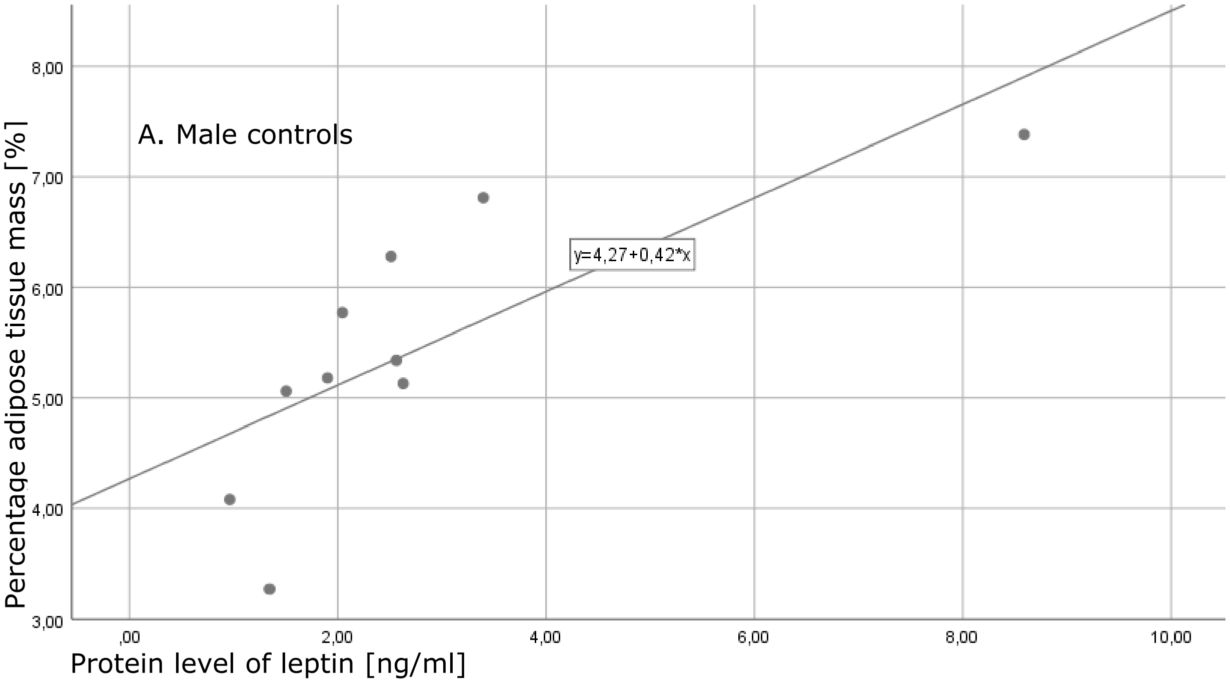

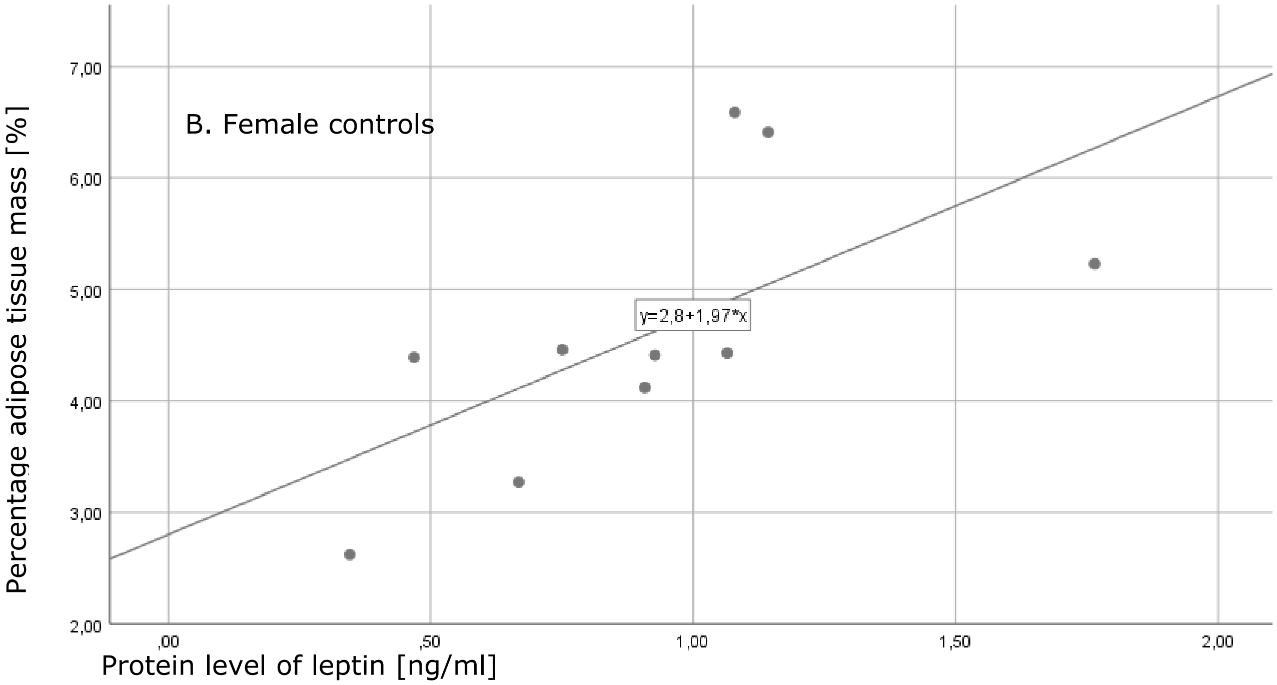

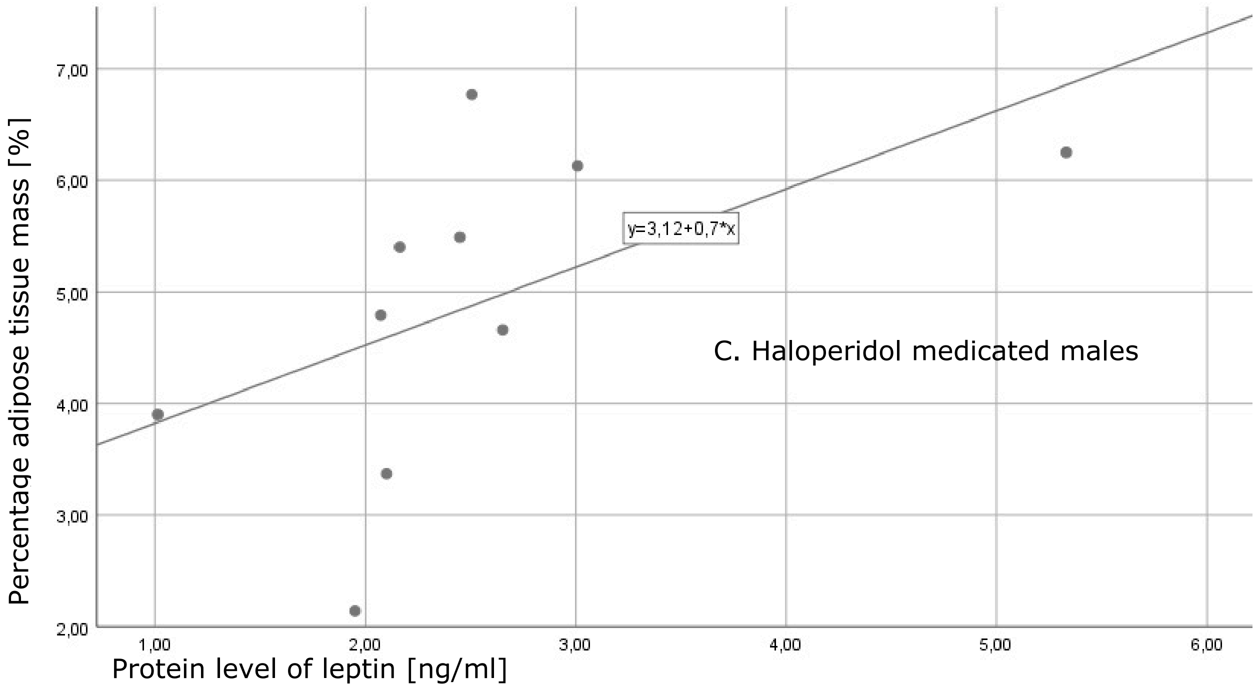

Percentage adipose tissue mass [%]
7,00
6,00
5,00
4,00
3,00
2,00
1,00
2,00
3,00
4,00
5,00
6,00
Protein level of leptin [ng/ml]
y=3,12+0,7*x
C. Haloperidol medicated males

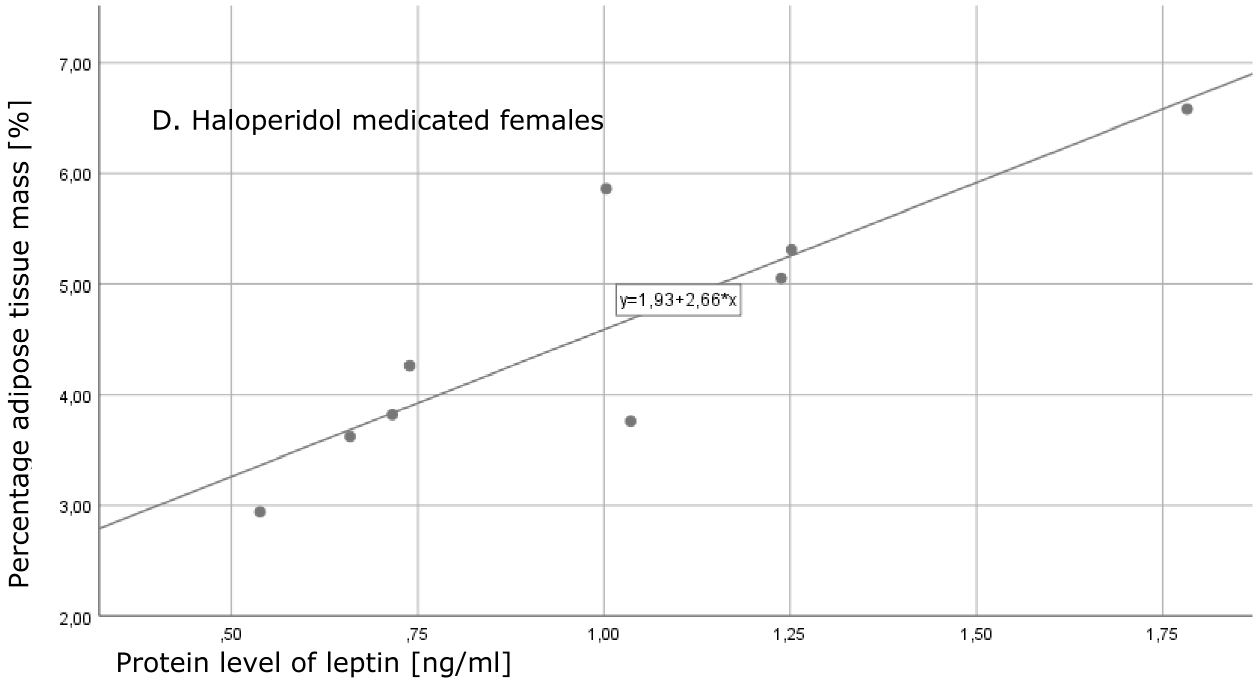

Percentage adipose tissue mass [%]
7,00
6,00
5,00
4,00
3,00
2,00
,50
,75
1,00
1,25
1,50
1,75
Protein level of leptin [ng/ml]
D. Haloperidol medicated females
y=1,93+2,66*x

E.-F. Nonparametric correlation of protein level ofadiponectin (abscissa) and percentage adipose mass of male and female haloperidol medicated SD rats.

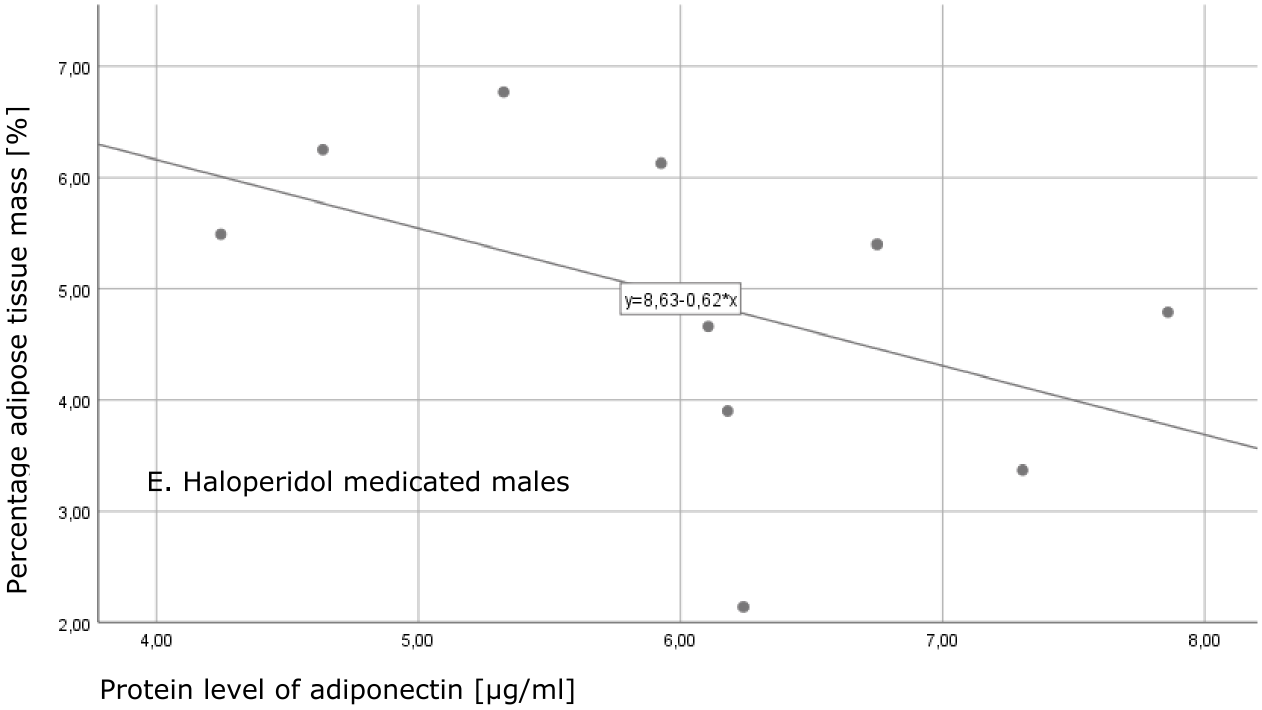

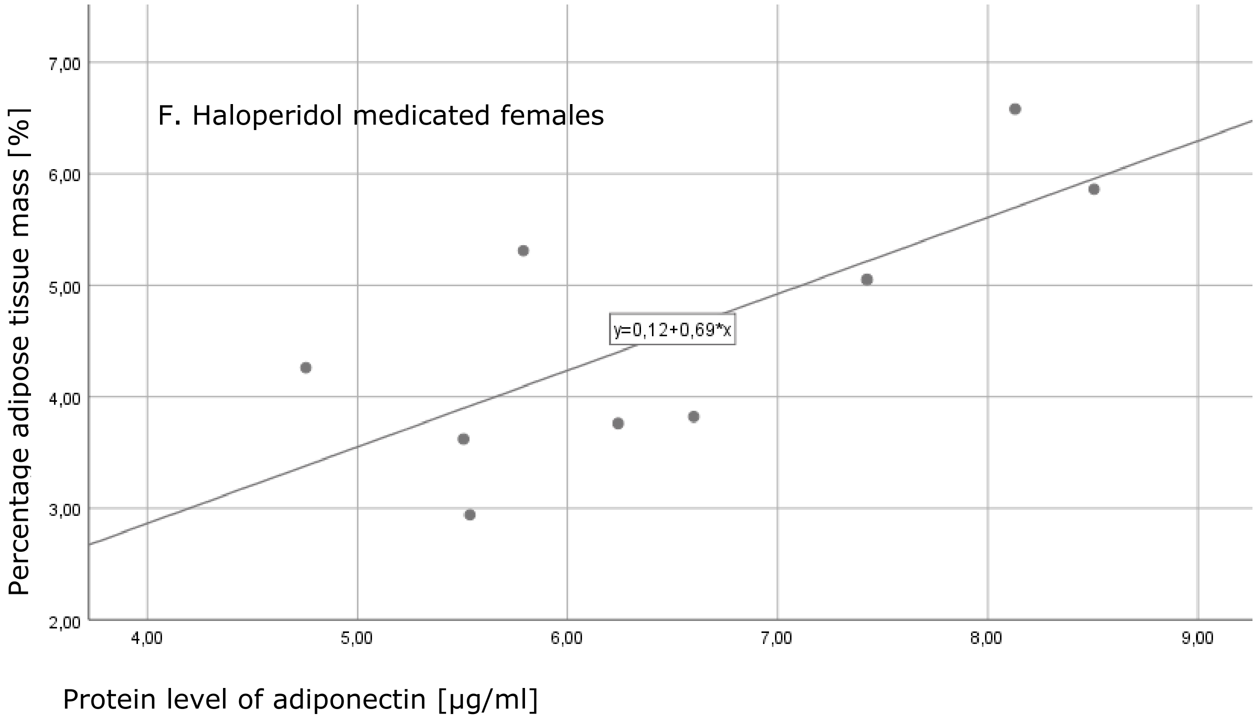

G.-H. Nonparametric correlation of HSL (abscissa) and PerilipinA (ordinate) of male and female control SD rats (HSL=hormon sensitive lipase).

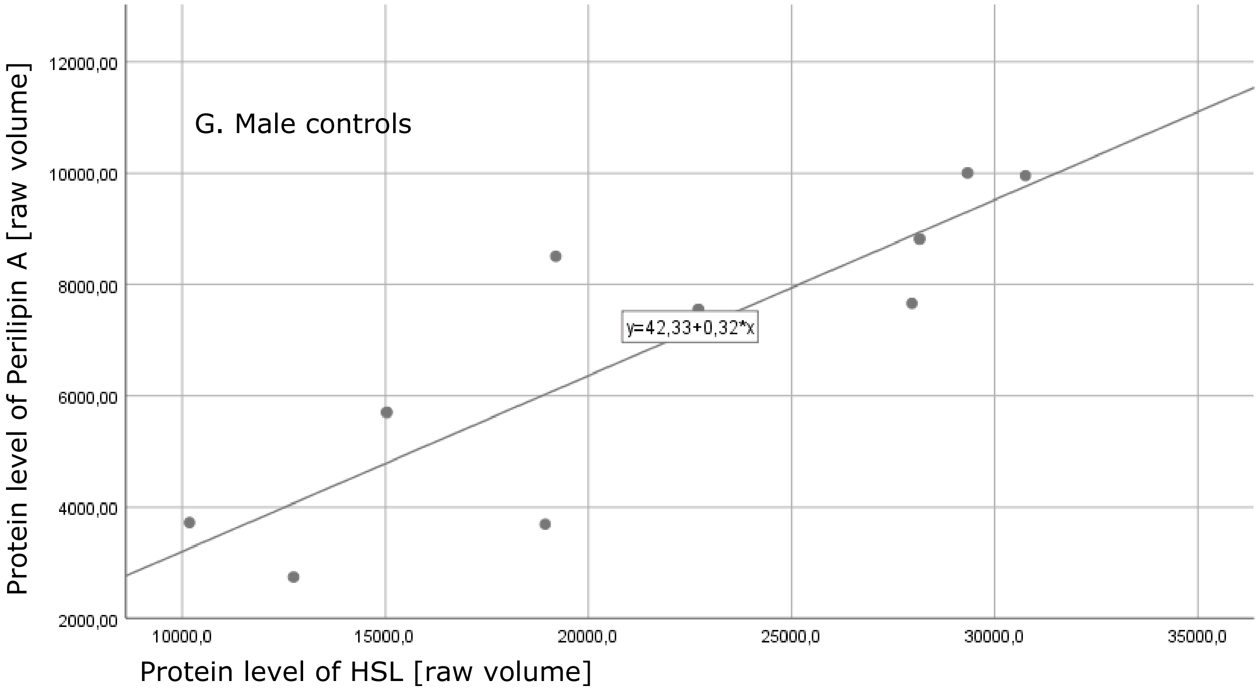

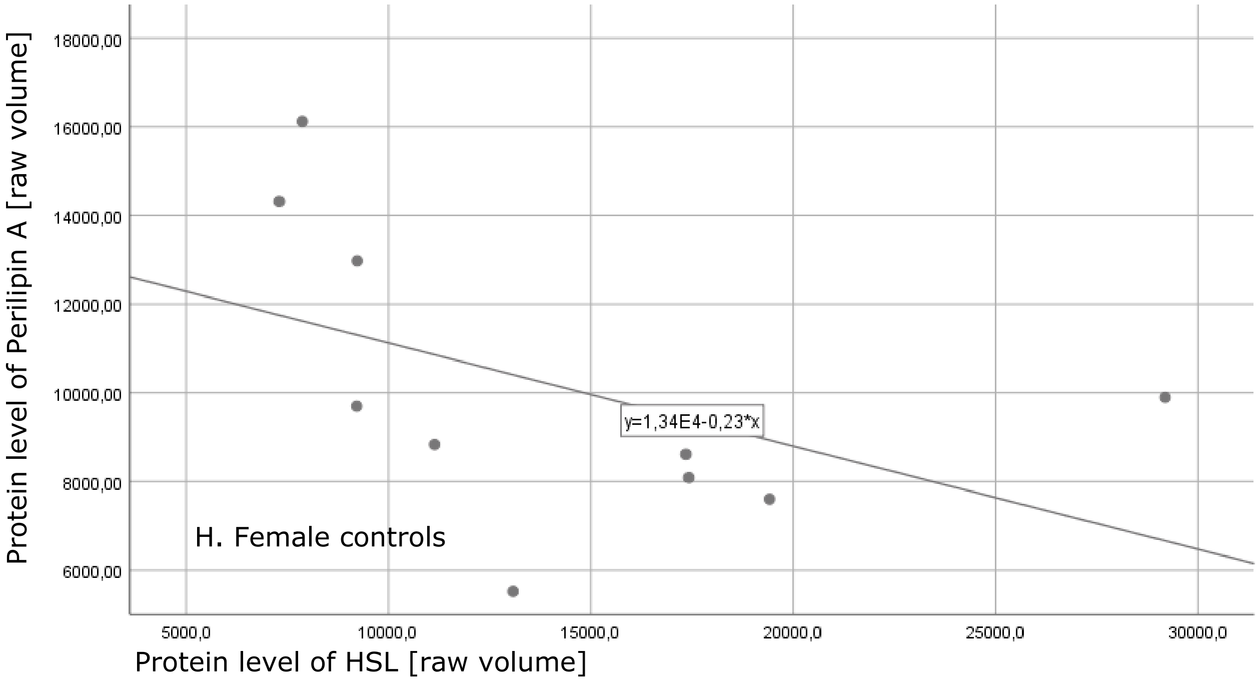

I. Nonparametric correlation of IR-ß (abscissa) and Glut-4 (ordinate) of female clozapine medicated SD rats (IR-ß= insulin receptor-ß; Glut-4= glucose transporter-4).

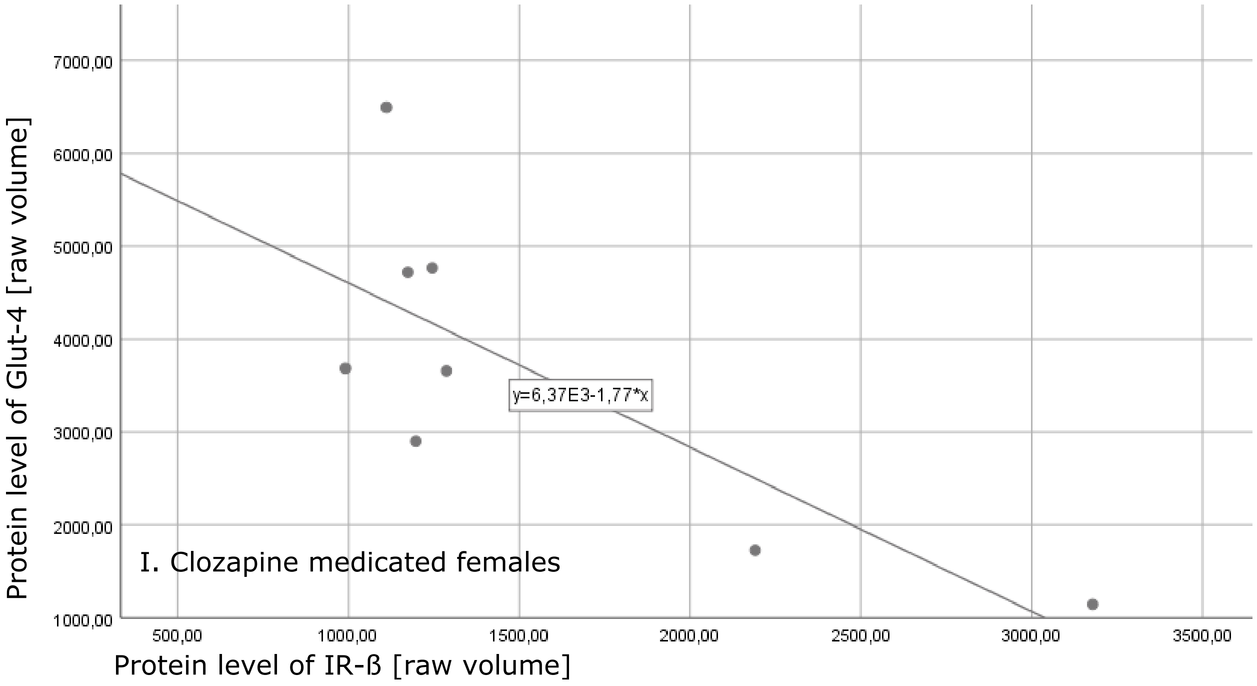

Figure S3

A. Final body weight (body weight gain over 12-weeks was shown in [23])

B Final body weight minus liver mass (liver mass was examined in [24])

C Final body mass minus adipocyte mass

D Final body weight minus liver and adipocyte mass

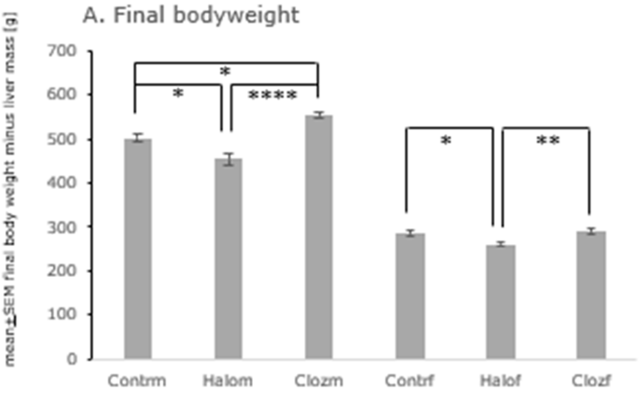

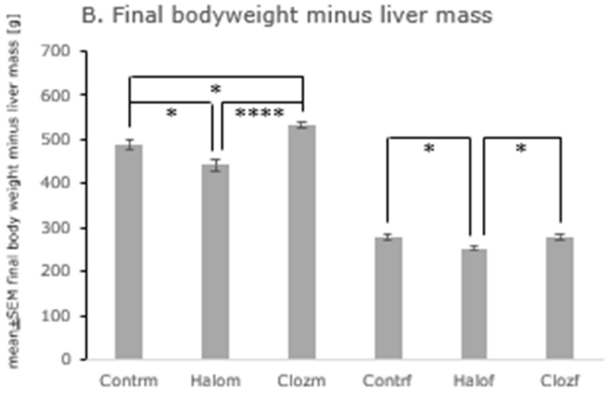

Two-way ANOVA showed differences for:

A. Final bodyweight

Medication[$F(2,54)=27.0$ $p \leq 0.000001$], SEX[$F(1,54)=1010.3$ $p \leq 0.000001$]and MEDICATIONxSEX [$F(2,54)=8.55$ $p=0.001$].

Posthoc-tests:

Higher final bodyweight in the male ($p=0.010$) clozapine treated group, lower final body weight in the male ($p=0.013$) and female ($p=0.021$) haloperidol group, and between haloperidol or clozapine treated males ($p=0.000003$) and females ($p=0.009$)

B. Final bodyweight minus liver mass

Medication[$F(2,54)=24.4$ $p \leq 0.000001$], SEX[$F(1,54)=997.1$ $p \leq 0.000001$]and MEDICATIONxSEX [$F(2,54)=7.7$ $p=0.001$].

Posthoc-tests:

Higher final bodyweight in the male ($p=0.018$) clozapine treated group, lower final body weight in the male ($p=0.016$) and female ($p=0.025$) haloperidol group, and between haloperidol or clozapine treated males ($p=0.000007$) and females ($p=0.014$)

C. Final bodyweight minus perirenal fat mass

Medication[$F(2,54)=29.5$ $p \leq 0.000001$], SEX[$F(1,54)=1226.8$ $p \leq 0.000001$]and MEDICATIONxSEX [$F(2,54)=8.3$ $p=0.001$].

Posthoc-tests:

Higher final bodyweight in the male ($p=0.010$) clozapine treated group, lower final body weight in the male ($p=0.007$) and female ($p=0.013$) haloperidol group, and between haloperidol or clozapine treated males ($p=0.000001$) and females ($p=0.006$)

D. Final bodyweight minus liver mass minus perirenal fat mass

Medication[$F(2,54)=26.4$ $p \leq 0.000001$], SEX[$F(1,54)=1216.3$ $p \leq 0.000001$]and MEDICATIONxSEX [$F(2,54)=7.4$ $p=0.001$].

Posthoc-tests:

Higher final bodyweight in the male ($p=0.021$) clozapine treated group, lower final body weight in the male ($p=0.008$) and female ($p=0.015$) haloperidol group, and between haloperidol or clozapine treated males ($p=0.000004$) and females ($p=0.010$)

* $p \leq 0.05$; ** $p<0.01$ **** $p<0.00001$

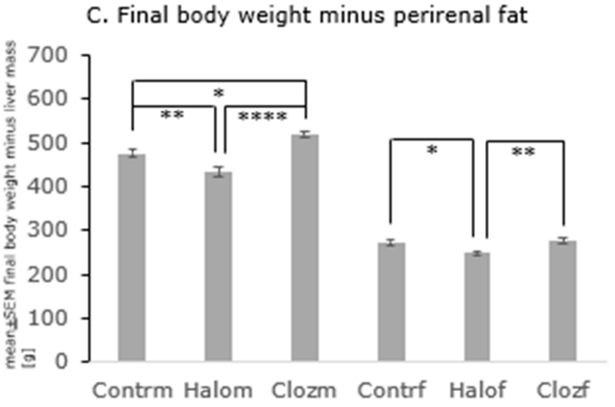

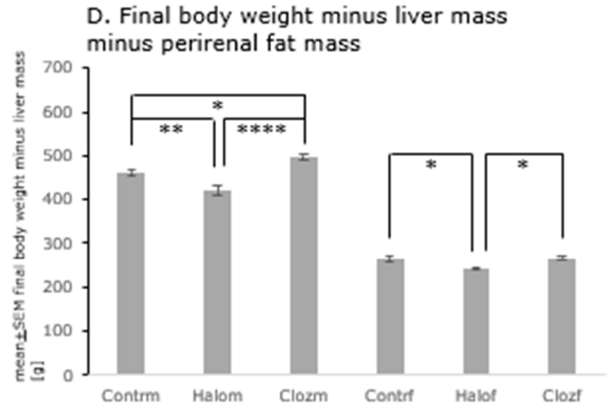

Supplement: Supplementary file 1 [file ijms-25-02188-s001.zip › ijms-2853426-supplementary.pdf]
